# Inhibition of the Transcription Factor PU.1 Suppresses Tumor Growth in Mice by Promoting the Recruitment of Cytotoxic Lymphocytes Through the CXCL9-CXCR3 Axis

**DOI:** 10.3390/cancers17162684

**Published:** 2025-08-18

**Authors:** Nichita Sleapnicov, Soon-Duck Ha, Shanshan Jenny Zhong, Jackie Duchscher, Sally Ezra, Shawn Shun-Cheng Li, Sung Ouk Kim

**Affiliations:** 1 Department of Microbiology & Immunology, Schulich School of Medicine & Dentistry, University of Western, London, ON N6G 2V4, Canada; 2 Department of Biochemistry, Schulich School of Medicine & Dentistry, University of Western Ontario, London, ON N6G 2V4, Canada

**Keywords:** PU.1 inhibition, DB2313, melanoma, tumor-associated macrophages, CXCL9

## Abstract

**Background**: Targeting tumor-associated macrophages (TAMs) is a promising immunotherapy for cancers, but current strategies are limited due to strategic caveats. PU.1 is a transcription factor required for macrophage generation and differentiation. To date, the effect of PU.1 inhibition on solid tumors is unknown. **Methods**: This study examines the anti-tumor effect of PU.1 inhibition and its mechanism using the small-molecule DB2313 in mouse melanoma and breast tumor models. **Results**: We found that inhibition of PU.1 by DB2313 suppresses B16-OVA melanoma and 4T1 breast tumor growth in mice. In the melanoma tumor model, DB2313 enhanced tumor recruitment of CD4^+^ T helper 1 (Th1) and cytotoxic T/natural killer (NK) cells by targeting TAMs. Transcriptome and targeted gene expression analyses revealed that PU.1 inhibition by DB2313 and small-interference RNAs enhances CXCL9 expression in bulk tumors, TAMs, and bone marrow-derived macrophages. The anti-tumor effects of DB2313 were abolished by depleting macrophages with clodronate or inhibiting the CXCL9-CXCR3 chemokine axis using CXCL9- or CXCR3-neutralizing antibodies. **Conclusions**: These results suggest that pharmacological inhibition of PU.1 suppresses tumor growth by at least promoting the infiltration of lymphocytes into tumors through the CXCL9-CXCR3 chemokine axis. Our study establishes a framework for developing TAM-modulating immunotherapies by targeting the transcriptional factor PU.1.

## Simple Summary

PU.1 is a critical transcription factor involved in the development and function of macrophages that play a central role in tumor initiation and progression. However, its role in tumor-associated macrophages (TAMs) remains poorly understood. This study investigates the impact of PU.1 inhibition using the chemical inhibitor DB2313 in a mouse tumor model. The findings aim to offer a potential therapeutic strategy for targeting PU.1 in TAMs to suppress tumor growth.

## 1. Introduction

Tumor establishment, growth, and responses to treatment are critically influenced by the tumor microenvironment (TME), which is composed of a variety of cell types, such as immune cells, endothelial cells, and cancer-associated fibroblasts, and by the tumor-TME crosstalk mediated by cytokines/chemokines, growth factors, and other mediators [1,2]. In particular, tumor-associated macrophages (TAMs), which often make up the largest portion of the immune cell populations within the TME, play a key role in determining the immunological characteristics of TME [3,4]. TAMs are heterogeneous, with both pro-inflammatory M1-like or anti-inflammatory M2-like phenotypes. M1-like TAMs prevent tumor establishment and growth by directly killing tumor cells through cytotoxic cytokines. However, these inflammatory cytokines can also support tumor growth and metastasis [1,5]. M1-like cells can be differentiated into M2-like cells, as well as multiple intermediate phenotypes, within the TME [3,4,5]. These heterogeneous TAMs largely promote tumor growth/metastasis and render resistance to anti-tumor immune therapies [6]. Hence, TAMs have been a potential target for anti-tumor therapies, and various strategies have been developed to prevent the generation of TAMs or re-programming them from tumor-supportive to tumor-suppressive phenotypes [7,8,9]. These TAM-targeted therapeutic strategies include depleting TAMs by targeting macrophage colony-stimulating factor receptors, inhibiting M2-signaling proteins such as phosphoinositide 3 kinase, Janus kinase 2, receptor-interacting protein kinase 1, or Bruton’s tyrosine kinase, and reprogramming TAMs by toll-like receptor agonists or CD40 ligands [7]. However, these approaches have been met with limited success [10] and other potential challenges [11,12,13], underscoring the need for novel strategies for targeting the TAM for immunotherapy.

PU.1, a member of the E26 transformation-specific (ETS) family transcription factor (TF), plays a key role in the early development of myeloid and lymphoid cells, as genetic PU.1 knockout mice lack myeloid cells and B cells [14,15] and are impaired in T cells [16] and NK cells [17]. In myeloid cells, a high level of PU.1 expression is required for their proper development, function, and differentiation [18,19,20], as well as for optimal expression of inflammatory cytokines [21,22,23]. In addition, PU.1 plays an important role in M2-like macrophage polarization [24,25]. While genetic studies employing complete or partial depletion of PU.1 underscores its crucial role in the development of immune cells, small-molecule inhibitors of PU.1, such as DB2313 (DB) and its analog DB1978, have shown anti-tumor effects in leukemia, where PU.1 is selectively required for their survival [26]. Notably, PU.1 inhibition showed minimal and reversible impacts on the development of lymphocytes and myeloid cells [26], suggesting that pharmacological inhibition of PU.1 is a viable strategy for regulating immune responses. However, to date, the effect of PU.1 inhibition on solid tumors and TAMs is yet to be explored.

To evaluate PU.1 as a potential therapeutic target for anti-cancer immunotherapy by reprogramming TAMs, we have investigated the effects and mechanisms of action of the PU.1 inhibitor DB in a mouse melanoma model. DB is a heterocyclic diamine derived from the clinically tested compound furamidine [27]. It has been shown to selectively inhibit PU.1 among ETS TFs by binding to the minor DNA groove adjacent to the major groove that comprises the ETS-binding core motif (5′-GGAA/T-3′) [26]. Using mouse melanoma and breast tumor models, we found that PU.1 inhibition by DB suppressed tumor growth, likely through promoting CXCL9 expression in TAMs, which enhances the tumor infiltration of lymphocytes, including CD4^+^ Th1, CD8^+^ cytotoxic T lymphocytes (CTLs), and NK cells.

## 2. Materials and Methods

### 2.1. Materials and Reagents

DB2313 (DB) was purchased from Glixx Laboratories (Hopkinton, MA, USA). For in vitro use, it was prepared in 500 mM stock solution in DMSO. For in vivo experiments, DB was prepared in 30% Polyethylene glycol 300 (PEG300; MediChemExpress; Monmouth Junction, NJ, USA) and 70% saline. Matrigel Basement Membrane Matrix was purchased from BD Biosciences (San Jose, CA, USA). InVivoMAb, rat IgG2a isotype control, anti-mouse CXCR3 (CD183), anti-mouse CXCL9 (MIG), and polyclonal Armenian hamster IgG isotype control antibodies were purchased from Bio X Cell (Lebanon, NH, USA). Antibodies used for flow cytometry and immunohistochemistry are listed in Supplementary Table S1. For immunoblotting analysis, PU.1 antibody (C-3) and anti-β-actin were purchased from Santa Cruz Biotechnology (Dallas, TX, USA) and Rockland (Limerick, PA, USA), respectively. The RNA protect reagent, RNeasy Mini Kit, and tissue shredder were purchased from Qiagen (Montreal, QC, Canada). The PureLinkTM RNA Mini kit and Lipofectamine RNAi MAX reagent were obtained from Invitrogen through Thermo fisher Scientific (Waltham, MA, USA).

### 2.2. Mice

C57BL/6 and BALB/c female mice aged 6–8 weeks were purchased from Charles River Laboratories Canada (Senneville, QC, Canada) and housed in the Animal Care and Veterinary Service facility at Western University (London, ON, Canada). Mice were housed under specific-pathogen-free conditions in groups of six animals; they were provided with a 12 h light–dark cycle, bedding and nesting materials, and food and water ad libitum. All efforts were made to minimize mice suffering and all mice experiments were conducted under the guidelines of approved animal care and use protocol (AUP2022-138), at Western University.

### 2.3. Cell Culture

B16-OVA melanoma cells (Dr. Tomoko Hayashi, UCSD; La Jolla, CA, USA) and the 4T1 breast cancer cell line (ATCC, Cat#; CRL-2539) were cultured in complete DMEM (Sigma-Aldrich Canada; Oakville, ON, Canada) supplemented with 10% fetal bovine serum and 100 U/mL penicillin–streptomycin (Thermo Fisher Scientific, Toronto, ON, Canada). G418 (200 µg/mL, WISENT INC; Saint-Jean-Baptiste, QC, Canada) was also added to cell culture to maintain OVA-expressing clones, but removed 24 h before inoculation. To prepare bone marrow-derived macrophages (BMDMs), bone marrow cells from the femurs of C57BL/6 mice (Charles River Laboratories Canada) were cultured in the presence of cell culture media (30%) obtained from L929-feeder cells producing macrophage colony-stimulating factor for 5–6 days, as previously described [28]. After washing off non-adherent cells with PBS, adherent cells were used as BMDMs.

### 2.4. Mouse Tumor Model

B16-OVA cells were harvested when they reached around 80% confluence after 3–4 passages and washed twice with serum-free DMEM, and 5 × 10^4^ cells were suspended with 50 µL of serum-free DMEM. The cell suspensions were mixed with 50 uL of Matrigel Basement Membrane Matrix and subcutaneously injected into the right hind flank of C57BL/6 mice (body weight; ~20 g) or the mammary fat pad of BALB/c mice. Mice were monitored daily, and drug injection was initiated when tumor volume reached around 50~60 mm^3^; mice were randomly divided into four or six groups (*n* = 5~6 per group) before treatment. DB2313 (DB; Glixx Laboratories, Hopkinton, MA, USA) were prepared in 30% PEG300 in saline, and 100 µL of vehicle (30% PEG300 and 70% saline) or DB (100 µL, 17 mg/kg) were injected every 2 days via intraperitoneal (IP) injection for 12 days (six doses).

For macrophage depletion experiments, mice were treated with i.p injection of 1 mg per mouse clodronate or control liposomes (LIPOSOMA BV; Amsterdam, The Netherlands) every 4 days, as described previously [29], with or without the vehicle or DB administration every 2 days for 12 days. All antibodies for mice injection were diluted in phosphate-buffered saline (PBS) to make experimental dosages, and Armenian hamster IgG isotype control (100 µL,140 µg/mouse, every 4 days, Clone; Polyclonal, Bio X Cell; Lebanon, NH, USA), anti-CXCR3 (100 µL, 140 µg/mouse, every 4 days, Clone; CXCR3-173, Bio X Cell), and anti-mouse CXCL9 (100 µL, 200 µg/mouse, every 3 days, Clone; MIG-2F5.5, Bio X Cell) were administered by i.p. injection. Tumor size was measured blindly using a caliper every 1–2 days in the morning, as indicated in the figure legend. Tumor volumes were calculated using formula V (mm^3^) = (W^2^ × L)/2 (V, volume; W, width; L, length), as previously reported [30], and are represented graphically. On the 13th day post initiation of treatment, all mice were euthanized with Isoflurane (5%) following the institutional guidelines, and tumor tissues were harvested from randomly selected three or four mice per group to study further. No apparent adverse events were observed in each experimental group.

### 2.5. Single-Cell Suspension Preparation from Tumors

Dissociation of tumors into single-cell suspension was prepared following the STEMCELL Technologies protocol. Briefly, tumor tissues were excised into millimeter-sized pieces using razor blades and digested in RPMI 1640 media containing collagenase/hyaluronidase (STEMCELL Technologies, Vancouver, BC, Canada) and DNase I (Sigma-Aldrich, St-Louis, MO, USA) for 30 min at 37 °C on a shaking platform. Tumor cell suspensions were passed with 70 μm filters and treated with RBC lysis buffer (BioLegend, San Diego, CA, USA) by following the manufacturer’s instructions. Single-cell suspensions were then washed with Phosphate-Buffered Saline (PBS) containing 2% FBS for further processing.

### 2.6. Tumor-Associated Macrophage (TAM) Isolation

F4/80 positive macrophages from tumors were isolated from single-cell suspensions using the PE positive selection kit (STEMCELL Technologies) and F4/80-PE antibody (Bio-Rad, Mississauga, ON, Canada) according to manufacturer instructions. Purified F4/80 positive cells were used for the RNA isolation, followed by RNA sequencing and quantitative real-time PCR (RT-qPCR).

### 2.7. Flow Cytometry

Single cells prepared from tumor tissues were counted, and 2–5 million cells were plated on a 96-well plate (VWR, round bottom) and washed with PBS. Cells were then incubated with PBS containing 1:8000 dilution of fixable viability dye (FVD) for 25 min in the dark at 4 °C. After washing twice with PBS, cells were incubated with FACS buffer containing 2% fetal bovine serum, monocyte block (Biolegend), Fc block (Biolegend), and surface-staining antibodies at room temperature for 25 min. Fluorescence minus one (FMO) control used the same amounts of monocyte block, Fc block, FVD, and surface-staining antibodies as samples, except for the antibody being controlled for. FMO controls for FVD were incubated in only PBS for the same timeframe.

For the intracellular staining, surface-stained cells were washed first with PBS and then incubated in fixation buffer (Invitrogen) for 20 min at room temperature. Cells were then washed twice with PBS and permeabilized using the permeabilization buffer (Invitrogen). Antibodies with intracellular targets were diluted in the permeabilization buffer and exposed to cells for 30 min in the dark at room temperature. FMO controls were also incubated in the permeabilization buffer. Cells were washed twice with PBS and suspended in 200 μL PBS or FACS buffer for FACS analysis.

The OVA peptide (SIINFEKL)-conjugated MHC-I tetramers were generated following the vendor’s protocol. Briefly, Flex-T™ H-2Kb monomers that harbor the peptide (Biolegend) were combined with PE-conjugated streptavidin (Biolegend) in the presence of D-biotin (50 mM, Sigma-Aldrich) and NaN3 (10%, Sigma-Aldrich) on ice overnight in PBS. After centrifuging down tetramers, they were resuspended and administered to samples in the same manner as the surface-stained antibodies.

### 2.8. Immunohistochemical (IHC) Staining

Tumor tissues were fixed with 10% formalin in PBS for 24 h and then paraffin-imbedded for IHC staining. Briefly, tumor tissues were dehydrated by sequential immersion once in 50%, 70%, 90%, and twice in 100% ethanol. The tissues were then immersed twice in xylene and dehydrated. Tissues were then paraffin-embedded, sectioned, and mounted on slides. For the IHC staining, tissue slides were deparaffinized in xylene, dehydrated by running through ethanol washes in reverse order, as described above, and stained with antibodies for CD8 and granzyme B. Secondary antibodies conjugated with HRP were used, and the signal was detected using the 3,3′-diaminobenzidine tetrahydrochloride substrate kit from Thermo Fisher Scientific. Counterstaining for nuclei was performed via hematoxylin staining. Images were taken at 100× magnification using a Qimaging cooled charged-coupled device camera on an Axioscope 2 (Carl Zeiss, Toronto, ON, Canada) microscope. The optical density of secreted Granzyme B was performed by using the color deconvolution tool on the Fiji-ImageJ (version 1.54m; converting DAB color into optical density using the following formula: OD = log_10_ (255/mean value)) [31]. CD8^+^ cells were quantified by cell-counting in the field of view (FOV), counting cells with a clear DAB color around the cell margins.

### 2.9. mRNA Sequencing and Transcriptomic Analysis

Total RNAs were prepared from TAMs using the Qiagen RNeasy kit (Qiagen, RNeasy Mini Kit) or PureLinkTM RNA Mini Kit (Invitrogen by Thermo Fisher Scientific) according to the manufacturer’s protocol. Tumor tissues were excised into ~30 mg pieces using razor blades and submerged into RNA to protect the tissue reagent (Qiagen) overnight at 4 °C. Tissues were then transferred to −80 °C and pulverized using a liquid nitrogen cold hammer. Total RNAs were then isolated using the Qiagen RNeasy Mini kit according to the manufacturer’s protocol. mRNA-enriched sequencing was performed by Genome Quebec (Montreal, QC, Canada) using an Illumina NovaSeq 6000 sequencer (San Diego, CA, USA; 2 × 25 million reads per sample). BAM files were generated by aligning sequences with NCBI37/mm9 mouse genome. The sequence datasets for TAMs and bulk melanoma are available in the Sequence Read Archive (SRA) repositories with accession numbers PRJNA1191843 and PRJNA1191505, respectively. Using these BAM files, read counts and fold of changes (FCs) for genes were calculated by using the FeatureCount tool using default settings [32] and the DESeq2 tool (c7 < 0.05) [33] in the Galaxy. The differential gene expression and gene counts were visualized in volcano plots and bubble heat maps using GraphPad Prism 10 and R Studio version 2024.04.0+735. Gene ontology and hallmark annotation analyses were performed in the genes changed (FC > 2, adj. *p* < 0.01) using the Metascape v3.5.20231212 [34]. For Gene Set Enrichment Analysis (GSEA) for bulk tumors, the Limma-voom tool version 3.58.1 [35] was used to filter out counts lower than 10 per million reads in the Galaxy, and the resulting differential expression tables were used for GSEA [36], followed by Cytoscape (version 3.10.3) visualization [37] with a node cut-off of 0.01 q-value and an edge cut-off of 0.5.

### 2.10. Transfection of Small Interfering (Si)RNA and LPS Stimulation in BMDMs

BMDMs were plated onto 6-well plates for overnight and transfected with mouse PU.1-specific siRNAs (Life Technologies, Carlsbad, CA, USA, Spi1 MSS277025 and Spi I MSS247676) for 18~20 h using Lipofectamine RNAiMAX (Invitrogen, Life Technologies), following the manufacturer’s instructions. Fresh culture media were then replaced and, after further culturing for 18 h, cells were activated by LPS (100 ng/mL) for 5 h. Total RNAs and cell lysates were prepared for RT-qPCR and Western blots, respectively.

### 2.11. RT-qPCR

Total RNAs were prepared using Qiagen RNeasy kit or Trizol (Ambion by Life Technologies), as described previously [38]. The quantity and quality of RNA were measured using a Thermo Scientific™ NanoDrop™ One Spectrophotometer. cDNAs were synthesized from the RNA preparations using 1 µg of total RNA and oligo dT_17_ primers with the M-MuLV reverse transcriptase (New England Biolabs, Ipswich, MA, USA), following the manufacturer’s protocol. Real-time quantitative PCR (qPCR) was conducted using the Universal Sybr Green Fast qPCR Mix (Ab Clonal, Wobum, MA, USA) in the Rotor-Gene RG3000 instrument (Montreal Biotech Inc, Montreal, QC, Canada) using the following primers: GAPDH: F: GCATTGTGGAAGGGCTCATG; R: TTGCTGTTGAAGTCGCAGGAG. PU.1: F: CTGGAACAGATGCACGTCC; R: CTGGTACAGGCGAATCTTTTTC. IL-1β: F: GTGGACCTTCCAGGATGAGG; R: GCTTGGGATCCACACTCTCC. CXCL9: F: AGGCACGGTCCACTACAAAT; R: TCCGGATCTAGGCAGGTTTG. Data were calculated relative to the levels of the glyceraldehyde 3-phosphate dehydrogenase (GAPDH) housekeeping gene.

### 2.12. Murine Cytokine/Chemokine Analysis

BMDMs (60,000 cells) in 96-well plates were treated with DB (500 nM) overnight and stimulated with LPS (100 ng/mL) for 18 h. Cell cultured media were collected after centrifugation at 3000× *g* for 10 min at 4 °C. The supernatants were then analyzed for IL-1β and CXCL9 (MIP) using the Mouse Cytokine/Chemokine Luminex platform, performed by Eve Technologies (Calgary, AB, Canada).

### 2.13. Immunoblotting

Immunoblotting was performed as previously described [38]. Briefly, BMDM cell lysates were prepared by lysing cells with an ice-cold lysis buffer containing 20 mM MOPS (pH 7.2), 2 mM EGTA, 5 mM EDTA, 1 mM Na3VO4, 40 mM β-glycerophosphate, 30 mM sodium fluoride, 20 mM sodium pyrophosphate, 0.1% SDS, 1% Triton X-100, and protease and phosphatase inhibitor tablets (Pierce, ThermoScientific, Rockford, IL, USA) for 10 min. Whole lysates were centrifuged at 14,000× *g* for 15 min at 4 °C, and supernatants were collected as total cell lysates. Proteins were denatured by boiling for 5 min in the presence of SDS-PAGE sample buffer (final concentrations of 5 mM Tris-HCl (pH 6.8), 0.1 M β-mercaptoethanol, 2% sodium dodecyl sulfate, 0.1% bromophenol blue, 10% glycerol), resolved in 10% SDS-polyacrylamide gels, and transferred onto 0.2 μm nitrocellulose membranes. The membranes were blocked at room temperature for 1 h with 5% (*w*/*v*) skim milk and immunoblotted with primary antibodies overnight at room temperature. After exposing corresponding secondary antibodies conjugated with horseradish peroxidase for 1 h at room temperature, bands were developed using the chemiluminescence reagent (BioRad Clarity Max Western ECL system), and images were detected using the BioRad Chemidoc XR^+^ System.

### 2.14. Statistics

Data were analyzed using GraphPad Prism Version 10.0 software, and the results are presented as scatter plots or bar graphs. Comparisons of means between groups were conducted with either Student’s *t*-tests or one-way ANOVA followed by Tukey’s multiple comparisons test.

## 3. Results

### 3.1. The PU.1 Inhibitor DB2313 (DB) Changes the Tumor-Infiltrated Immune Cell Repertoire and Suppresses Tumor Growth in a Melanoma Mouse Model

**Figure 1:**
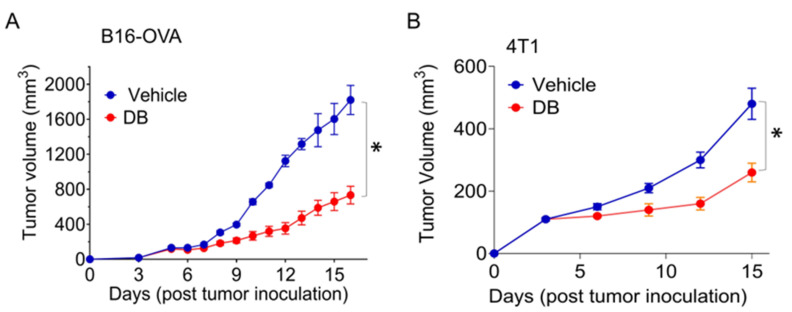
**DB suppresses tumor growth in melanoma and breast mouse tumor models.** Murine melanoma B16-OVA (**A**) and mammary tumor 4T1 (**B**) cells (5 × 10^4^) were injected into the right hind flank or mammary fat pad of C57BL/6 and BALB/c mice, respectively. Peritoneal injections of the PU.1 inhibitor DB2313 (DB; 17 mg/kg, every 2 days) were started when tumor volume reached around 50~60 mm^3^ (**A**) or on day 3 (**B**) for 12 days. Error bars are expressed as mean ± s.d (*n* = 6). Statistical significance was determined using Student’s *t*-test (*, *p* < 0.002).

To investigate the impact of PU.1 inhibition on solid tumors, we utilized the PU.1 inhibitor DB in the B16-OVA melanoma and 4T1 breast tumor models. After 4–5 days of tumor cell inoculation, DB was administered via intraperitoneal injection at a dose of 17 mg/kg every two days, which did not affect overall leukocyte generation and survival [26]. Based on tumor volume, DB significantly suppressed ~75% and 50% of tumor growth in both melanoma and breast tumors, respectively (Figure 1).

**Figure 2:**
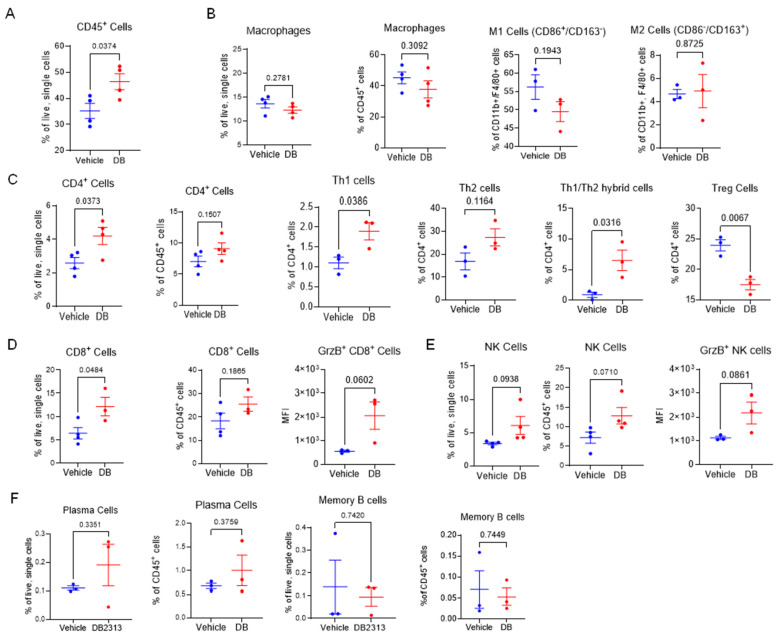
**DB recruits cytotoxic immune lymphocytes in melanoma tumors.** (**A**) Flow cytometry measurement of frequencies of CD45^+^ cells as a percentage of live single cells between DB- and vehicle-treated tumors. (**B**) Relative frequencies of macrophages (CD45^+^, CD11b^+^, F4/80^+^) as a % of live single cells or CD45^+^ immune cells, and subtypes M-1 (CD86^+^, CD163^−^) and M-2 (CD86^−^, CD163^+^) as a % of macrophages between vehicle- and DB-treated tumors. (**C**) Relative frequencies of CD4^+^ T cells (CD45^+^, CD3^+^, CD4^+^) and subtypes Th1 (Foxp3^−^,Tbet^+^, GATA3^−^), Th2 (Foxp3^−^, Tbet^−^, GATA3^+^), Tregs (Foxp3^+^ CD25^+^), and Th1/Th2 (Foxp3^−^, Tbet^+^, GATA3^+^) between DB- and vehicle-treated tumors. (**D**) Relative frequencies of CD8^+^ T cells (CD45^+^, CD3^+^, CD8^+^, NK1.1^−^). Cytotoxic CD8^+^ T cells were identified via expression of granzyme B, as measured by median fluorescence intensity (MFI). (**E**) Relative frequencies of total and cytotoxic NK cells (CD3^−^, NK1.1^+^, GrzB^+^). (**F**) Relative frequencies of plasma cells and B cells between DB- and vehicle-treated tumors. Statistical analysis by student’s unpaired *t*-tests; significance defined as *p* < 0.05 (*n* = 3–4).

Since tumor-associated immune cells play a key role in determining tumor growth, we focused on the B16-OVA melanoma and enumerated immune cells infiltrating the tumors in DB-treated mice in comparison to vehicle treatment. Overall, DB recruited more hematogenous immune cells (CD45^+^; ~50% of live cells isolated) into the tumors, compared to ~35% in vehicle-treated tumors (Figure 2A). Using FACS with an appropriate gating strategy (Supplementary Figure S1), we profiled tumor-associated immune cells, including macrophages, T cells, B cells, and NK cells. Within the CD45^+^ cell population, no significant changes were detected in the ratios of F4/80^+^/CD11b^+^ macrophages relative to the total number of cells or CD45^+^ cells (Figure 2B). Additionally, within the macrophage population, the ratio between M1 (CD86^+^/CD163^−^) and M2 (CD86^−^/CD163^+^) macrophage subtypes remained unchanged. These findings suggest that DB treatment does not significantly affect the recruitment or polarization of macrophages. In contrast, significant changes in both CD4^+^ and CD8^+^ T cells were observed in tumors treated with DB compared to vehicle control. Specifically, a significantly higher ratio of CD4^+^ T cells, relative to the total tumor cells (but not CD45^+^ cells), was detected in DB-treated tumors (Figure 2C). Within the CD4^+^ T cell population, the ratios of Th1 (T-bet^+^) and Th1/Th2 hybrid cells [39] were significantly increased, whereas the ratio of Th2 (Gata^+^) cells remained unchanged following DB treatment. In contrast, the regulatory T (Treg; FoxP3^+^) cell population was significantly decreased by DB treatment (Figure 2D, far right panel). The ratios of CD8^+^ T cells relative to the total live cells were substantially increased in DB-treated tumors, and the ratios of CD8^+^/granzyme B^+^ (GnzB^+^) cytotoxic T cells tended to be higher in DB-treated tumors (Figure 2D, left two panels), although this increase did not reach statistical significance (Figure 2D, right panel). Moreover, NK cell and GnzB^+^-NK cell populations tended to be higher in DB-treated tumors without reaching statistical significance (Figure 2E). In comparison, the ratios of plasma B cells and memory B cells remained unchanged by DB treatment (Figure 2F). Collectively, these data suggest that DB treatment enhances infiltration of anti-tumor Th1 (CD4^+^) and CD8^+^ cytotoxic T cells, while decreasing infiltration of Treg cells, into tumors.

### 3.2. Depletion of Tumor-Associated Macrophages (TAMs) Abolished the Anti-Tumor Effects of DB

**Figure 3:**
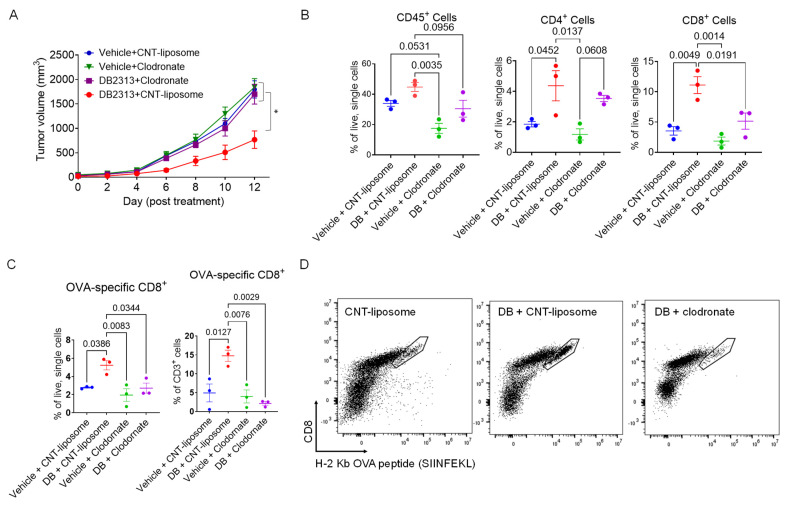
**Macrophage depletion inhibits DB-induced T cell recruitment to the tumor.** (**A**) Macrophage depletion abolished the anti-tumor effect of DB. Shown are growth curves of B16-OVA tumors) treated with vehicle or DB (DB, 17 mg/kg), together with either control liposome (1 mg/mouse) or clodronate (1 mg/mouse). Statistical significance was determined using one-way ANOVA with Tukey’s multiple comparisons test (n = 5–6; *, *p* < 0.05). (**B**) Relative frequencies of immune cells (CD45^+^), CD4 T cells (CD45^+^/CD3^+^/CD4^+^), and CD8 T cells (CD45^+^/CD3^+^/CD8^+^) as a percentage of live, single cells, measured by flow cytometry in the various treatment groups. (**C**) Relative frequencies of CD8^+^/OVA-tetramer^+^ cells corresponding to the different treatment groups. (**D**) Representative FACS plots (8000 events in CD3^+^/NK1.1^−^ gate; boundary lines indicate OVA-specific CD8^+^ cells). Statistical significance was tested by one-way ANOVA with Tukey’s multiple comparisons test, with significance defined as *p* < 0.05 (*n* = 3).

Given that macrophages play a crucial role in the recruitment of T cells to tumors and that PU.1 expression is typically low (or absent) in T cells but high in macrophages, the observed effect of DB in enhancing T cell recruitment to tumors may be mediated by macrophages. To investigate this possibility, we examine the anti-tumor effects of DB in B16-OVA tumor-bearing mice with or without macrophage depletion using clodronate. Treatment with clodronate liposomes led to ~50% reduction in TAMs compared to the control liposome (Supplementary Figure S2). Notably, clodronate significantly reversed the tumor growth-suppressing effect of DB, whereas clodronate or the control liposome alone had no effect (Figure 3A). In line with these results, flow cytometry analysis showed that the enhanced recruitment of CD45^+^ immune cells, as well as CD4^+^ and CD8^+^ T cells into tumors by DB, was substantially diminished when macrophages were depleted (Figure 3B). To further examine if DB enhanced recruitment of tumor-specific cytotoxic T cells in a macrophage-dependent manner, we used tetramers of H-2 Kb (C57BL6 mouse MHC-I molecule) linked to the OVA peptide (SIINFEKL). DB significantly increased the recruitment of OVA-specific CD8^+^ T cells into tumors by more than two-fold per given number of live tumor cells, as well as that of CD3^+^ lymphocytes (Figure 3C). Importantly, this increase in OVA-specific CD8^+^ T cell recruitment was no longer observed when TAMs were depleted (Figure 3C,D). These results suggest that TAMs play a key role in DB-induced recruitment of cytotoxic T cells and the suppression of melanoma growth.

### 3.3. DB Selectively Enhances the Expression of CXCL9 mRNA in Melanoma TAMs in Vivo

**Figure 4:**
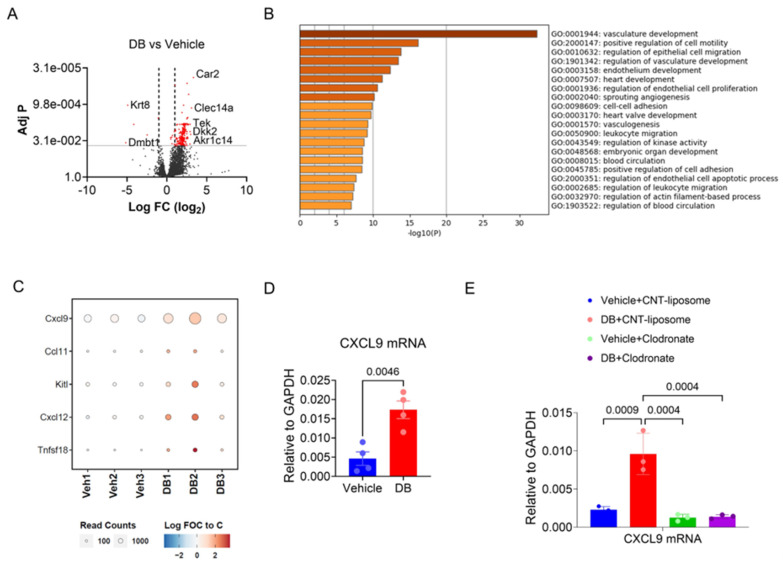
**DB enhances the expression of CXCL9 mRNA in tumor-associated macrophages (TAMs).** (**A**) Volcano plot showing differentially expressed genes between TAMs isolated from DB- or vehicle-treated melanoma tumors. Red dots indicate genes with an adj. *p* < 0.01. (**B**) DB-affected genes (FC > 1.5, adj. *p* < 0.05) were analyzed for GO terms using the Metascape online program. The top 20 GO and hallmark terms are presented. (**C**) Bubble heatmap showing five cytokines displaying increased expression in DB-treated TAMs compared to vehicle-treated TAMs. (**D**) CXCL9 mRNA levels in TAMs isolated from DB- or vehicle-treated tumors were determined by real-time PCR. (**E**) TAM depletion by clodronate abolished the effect of DB on CXCL9 mRNA expression. Shown are the mRNA levels of CXCL9 (relative to GAPDH) in tumors under the specified treatments. Statistical significance was determined by one-way ANOVA followed by Tukey’s multiple comparisons test (*n* = 3–4).

To confirm the effect of DB on TAMs, F4/80^+^ macrophages were isolated (Supplementary Figure S3) from tumors of mice 13 days after treatment with vehicle or DB (three mice in each group). Transcriptomic analysis of the isolated TAMs identified 259 genes that were significantly altered (>2-fold, *p* < 0.05) using DB treatment, including 255 genes up-regulated and four down-regulated genes (Figure 4A). Metascape analysis of the 255 up-regulated genes identified vasculature development, positive regulation of cell motility, and leukocyte migration as the top-ranked GO terms (Figure 4B). Among the five cytokine/chemokine genes induced by DB (Figure 4C), the transcript for CXCL9 showed the largest increase in expression in DB-treated TAMs compared to vehicle-treated TAMs, with a 2.1-fold increase (*p* < 0.01; Supplementary Table S2). This increase in CXCL9 mRNA expression in DB-treated TAMs was subsequently confirmed by qPCR (Figure 4D). Importantly, the increase in CXCL9 mRNA levels in DB-treated tumors was abolished when macrophages were depleted using clodronate (Figure 4E). Collectively, these results suggest that DB elevates CXCL9 production within the TME via TAMs.

### 3.4. DB and PU.1 Knocking Down Enhances CXCL9, but Inhibits IL-1β mRNA Expression in LPS-Stimulated BMDMs

**Figure 5:**
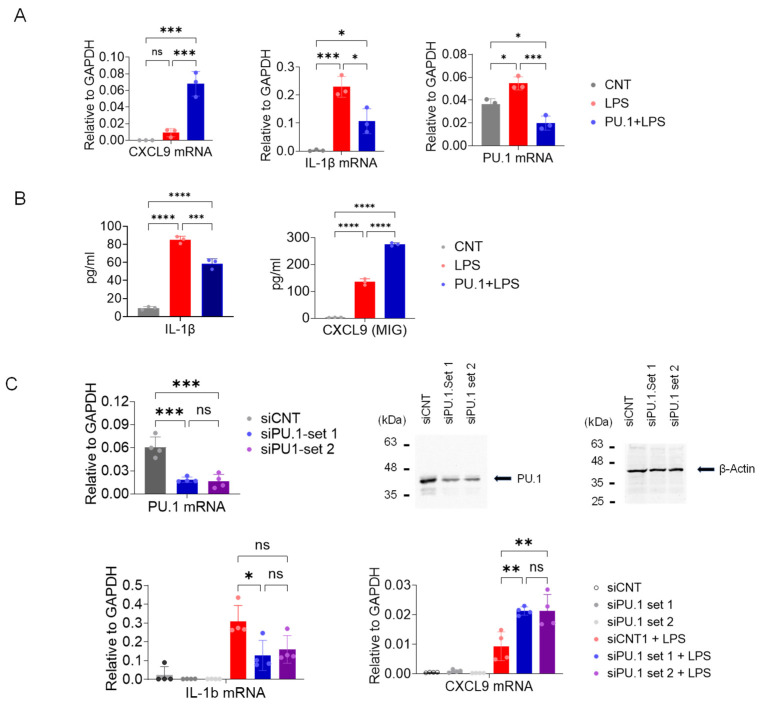
**Inhibition of PU.1 by DB and siRNAs enhances CXCL9 transcription in BMDMs**. (**A**,**B**) Macrophage colony-stimulating factor-derived BMDMs were exposed to the drug vehicle or DB (500 nM) for 18–20 h and then activated by LPS (100 ng/mL, 5 h (**A**) or 18 h (**B**)). (**A**) RNAs from BMDMs were isolated and mRNA expression of CXCL9, IL-1β, and PU.1 was analyzed by RT-qPCR. (**B**) Production of IL-1β and CXCL9 was measured from the cell culture media using the Luminex platform. Statistical significance was determined by one-way ANOVA followed by Tukey’s multiple comparisons test (*n* = 3; * *p* ≤ 0.05, ** *p* ≤ 0.01, *** *p* ≤ 0.001). (**C**) Similarly, BMDMs treated with control or PU.1-targeting siRNAs were activated by LPS for 5 h. Top left panel: PU.1 mRNA levels (relative to GAPDH) from the same cells, determined by RT-qPCR. Top right panels: Western blot of total cell lysates of BMDMs transfected with PU.1-specific siRNA or random control siRNA (siCNT). β-actin was included as a loading control. Bottom panels: Expression levels of CXCL9 and IL-1β in BMDMs transfected with PU.1-specific or control siRNA, with or without LPS treatment. * adj. *p* ≤ 0.05, ** *p* ≤ 0.01, *** *p* ≤ 0.001, **** *p* ≤ 0.0001, one-way ANOVA test followed by Tukey’s multiple comparisons test (*n* = 3).

Unexpectedly, the above data suggests that PU.1 inhibition enhanced the expression of CXCL9 in TAMs. Since TAMs are partially derived from monocytes recruited to the tumor [5], we examined the role of PU.1 in expression of CXCL9 in BMDMs stimulated with 100 ng/mL lipopolysaccharide (LPS) for 5 h. Consistent with data shown in Figure 4, DB enhanced expression of CCXL9 mRNA (Figure 5A, left panel). Since PU.1 is required for the optimal expression of IL-1β [22] and PU.1 expression is autoregulatory in part [40], we examined the expression of these genes with DB-pretreat BMDMs. As expected, DB substantially suppressed the transcription of these genes (Figure 5A). Aligning with mRNA data, the production of IL-1βand CXCL9 cytokines was also inhibited and enhanced, respectively, by DB in response to LPS stimulation (Figure 5B and Figure S5). Similarly, knocking down PU.1 by two different siRNAs (Figure 5C, top panels) enhanced CXCL9, but inhibited IL-1β transcriptions in BMDMs (Figure 5C, bottom panels).

### 3.5. The CXCL9-CXCR3 Chemokine Axis Plays a Key Role in the Recruitment of Cytotoxic Lymphocytes into Tumors and the Tumor Growth Suppression Induced by DB

**Figure 6:**
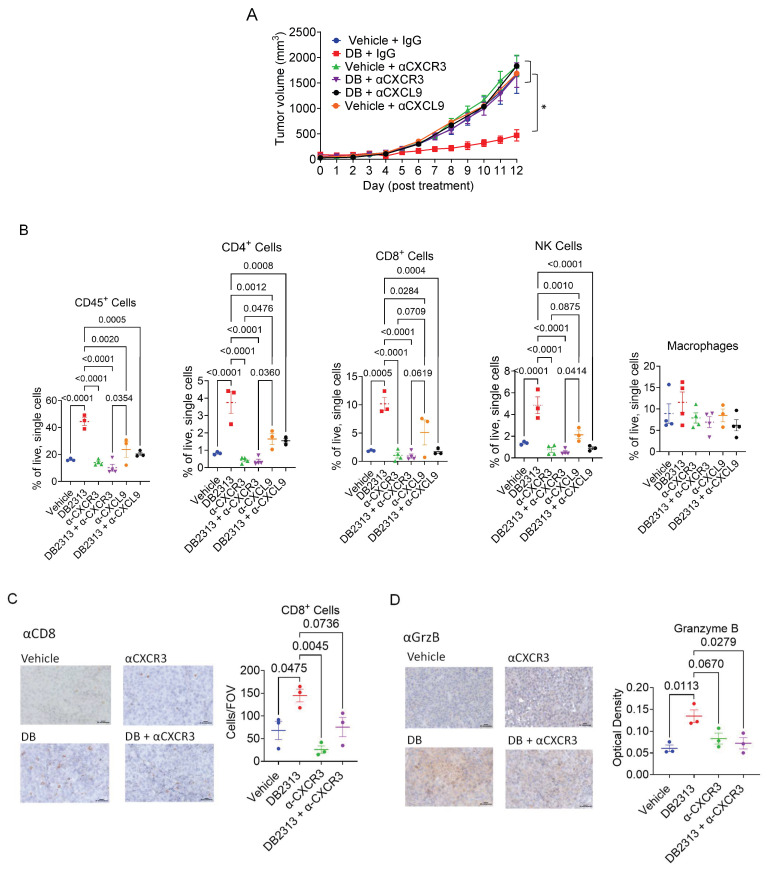
**CXCL9- and CXCR3-neutralizing antibodies abolish the anti-tumor effect of DB by reducing TILs**. (**A**) Growth curves of B16-OVA melanoma treated with DB or vehicle (every 2 days) in combination with neutralizing antibodies for CXCL9 (αCXCL9, every 3 days), CXCR3 (αCXCR3, every 4 days), control IgG (every 4 days). (**B**) Relative frequencies of immune cells (CD45^+^), CD4 or CD8 T cells (CD45^+^/CD3^+^, CD4^+^ or CD8^+^), and NK cells (CD45^+^/CD3^−^/NK1.1^+^). Macrophages (CD45^+^/CD11b^+^/F4/80^+^) were included for comparison. (**C**,**D**) Immunohistochemical staining of CD8 and Granzyme B (GrzB) in tumors using the specified treatments. Tumor tissues were stained with CD8^−^ and GrzB-specific antibodies, followed by incubation with HRP-conjugated secondary antibodies. HRP signals were detected using the DAB substrate kit, as described in Materials and Methods. The corresponding quantification data are presented in the right panels of (**C**,**D**); CD8 immunohistochemistry (IHC) is quantified as cells per field of view (cells/FOV), and Granzyme B (GrzB) IHC is quantified by the optical density of DAB staining. Statistical significance was assessed using a one-way ANOVA followed by Tukey’s multiple comparisons test. *p* < 0.05. Sample sizes: *n* = 5–6 for panel (**A**); *n* = 3–4 for other panels.

While our study demonstrates that DB increases CXCL9 expression in BMDMs and TAMs, effective T cell recruitment into tumors requires the CXCL9 receptor, CXCR3, expressed on T cells. Since the CXCR3-CXCL9 axis plays a critical role in tumor infiltration and reinvigoration of CD8^+^ T cells in response to PD-1 blockade [41], we next examined the role of CXCL9 and CXCR3 in mediating the anti-tumor effects of DB using the B16-OVA tumor model. Specifically, tumor-bearing mice were treated with DB together with control IgG, or neutralizing antibodies for CXCR3 (αCXCR3, 140 µg/mouse) every 4 days or CXCL9 (αCXCL9, 200 µg/mouse) every 3 days, starting 6 days after B16-OVA cell inoculation. While αCXCR3 and αCXCL9 alone did not affect tumor growth in vehicle-treated mice, the tumor-suppressive effect of DB, observed in DB + control IgG-treated mice, was abolished in mice treated with DB in combination with either αCXCR3 or αCXCL9 (Figure 6A). Mechanistically, αCXCR3 or αCXCL9 treatment significantly blocked the recruitment of CD45^+^ immune cells, CD4^+^/CD8^+^ T cells, and NK cells, but not macrophages, into tumors induced by DB (Figure 6B). In line with these data, immunohistochemistry analysis of tumor tissues confirmed that DB enhanced recruitments of CD8^+^ cells into tumors (Figure 6C) and production of GrzB in tumors (in both intracellular and extracellular forms; Figure 6D), both of which were inhibited by αCXCR3.

### 3.6. The CXCL9-CXCR3 Chemokine Axis Is Responsible for the DB-Induced Global Transcript Changes That Promote Anti-Tumor Immune Responses

**Figure 7:**
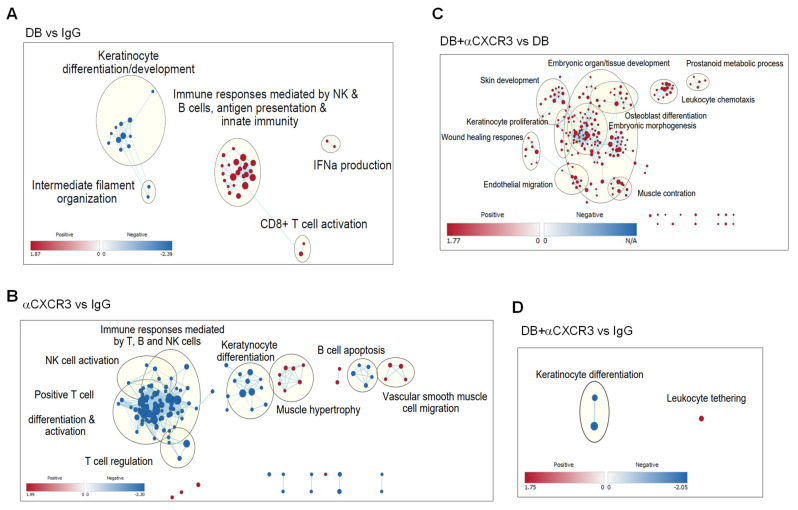
**The CXCL9-CXCR3 axis is responsible for DB-induced transcriptional changes in tumors.** B16−OVA tumors under the same treatments described in Figure 6A were harvested on day 13 for mRNA sequencing. Transcript counts were analyzed by GSEA and were visualized using the Cytoscape program. Enriched GO nodes (dots) and leading edge (grouped in circles with key functional annotation) are presented in pair-wise comparisons (up-regulation in red, down-regulation in blue), including DB + IgG vs. Vehicle + IgG (**A**), Vehicle + αCXCR3 vs. Vehicle + IgG (**B**), DB + αCXCR3 vs. DB + IgG (**C**), DB + αCXCR3 vs. Vehicle + IgG (**D**).

To examine the role of the CXCL9-CXCR3 chemokine axis in global transcript changes in tumors, total mRNAs extracted from the whole tumor tissues were sequenced, and the differential expression of transcripts was analyzed using Gene Set Enrichment Analysis (https://www.gsea-msigdb.org/gsea/index.jsp; accessed on 9 April 2024). The data were then visualized using the Cytoscape platform (Gene Ontology Biological Process dataset; FDR < 0.5, https://cytoscape.org/; accessed on 9 April 2024). Global transcripts of DB-treated tumors were highly enriched in genes related to both innate and adaptive immune responses, while genes associated with nonimmune functions, such as cell development and differentiation, were suppressed (Figure 7A; node list and NES in Supplementary Table S3). Compared to control IgG-treated tumors, αCXCR3 treatment largely down-regulated GO and hallmark terms related to lymphocyte immune responses, such as T, B, and NK cell activation and differentiation. In contrast, genes associated with nonimmune cell function, such as keratinocyte and vascular cell growth and migration, were up-regulated (Figure 7B; Supplementary Table S3). When comparing the transcriptomics of DB + αCXCR3 with those of DB-treated tumors, we observed substantial enrichments of various GO/hallmark terms related to nonimmune cell proliferation/differentiation, as well as leukocyte chemotaxis (Figure 7C; Supplementary Table S3). Also, when comparing the transcripts of DB + αCXCR3-treated tumors with those of IgG-treated tumors, most of the DB-enriched GO/hallmark terms were absent (Figure 7D; Supplementary Table S3). Altogether, these data suggest that CXCR3 plays a key role in the global enrichment of immune response gene transcription in DB-treated tumors.

## 4. Discussion

PU.1 is a member of the ETS family of transcription factors, which are considered oncogenic [42]. Previous studies on leukemia have shown that PU.1 can be either oncogenic [26,43,44] or tumor-suppressive [45,46] depending on the type of leukemia. In solid tumors, however, high expression levels of PU.1 have been associated with shorter survival rates across different cancers (Supplementary Figure S4), including melanoma and breast cancer, suggesting a pro-tumor role for PU.1. [47]. In this study, we show that pharmacological inhibition of PU.1 by DB significantly suppressed tumor growth in the B16-OVA and 4T1 mouse tumor models. Our in-depth investigation using the B16-OVA melanoma model revealed that the anti-tumor effect of DB is mediated by enhanced tumor infiltration of cytotoxic lymphocytes, particularly cytotoxic CD8^+^ T cells, which depends on the increased expression of CXCL9 by tumor-associated macrophages (TAMs) and the CXCL9-CXCR3 chemokine axis.

TAMs are the most abundant immune cells in the tumor microenvironment of many tumors, and play crucial roles in tumor development [3,4,48,49]. We also found that TAMs comprise approximately 40% of the hematogenous immune cells in our melanoma mouse model (Figure 2B). The crucial involvement of TAMs in mediating the antitumor effect of DB was substantiated by macrophage depletion experiments, which showed a nearly complete abatement of the DB’s tumor-suppressive effect (Figure 3A). Moreover, the enhanced recruitment of CD45^+^ immune cells, CD4^+^ and CD8^+^ T lymphocytes, and OVA-specific CD8^+^ cells induced by DB were significantly abrogated by macrophage depletion (Figure 3B,C). Since TAMs modulate TME mainly by releasing cytokines and chemokines, we examined how PU.1 inhibition (by DB and siRNAs) affects the expression of cytokines/chemokines in LPS-activated BMDMs. LPS potently activates the toll-like receptor 4 (TLR4), which is activated in TAMs and other cells within tumors and plays a key role in modulating TME [50], including in melanoma [51]. In BMDMs, LPS induced expression of both CXCL9 and IL-1β mRNAs (Figure 5). As expected for the transactivating role of PU.1, DB and knocking down PU.1 suppressed the expression of Il1b [52]. However, PU.1 inhibition (either through DB- or PU.1-targeting siRNAs) further enhanced the expression of CXCL9. Given that the chemokine is involved in the recruitment of cytotoxic lymphocytes into tumors through binding to the common receptor CXCR3 [53], thereby suppressing tumor growth, our finding that DB specifically enhanced the expression of CXCL9 underscores the importance of the CXCL9-CXCR3 axis in mediating the anti-tumor effect of DB. Our data also demonstrates that the source of CXCL9 transcription is TAMs, as the increase in CXCL9 transcript expression by DB was completely abrogated when macrophages were depleted (Figure 4E). Nevertheless, the exact mechanism underlying how DB enhances CXCL9 expression in BMDMs and TAMs is unknown. Our findings are also at odds with a report showing that PU.1 depletion inhibited the expression of CXCL9/10/11 in interferon-γ-activated microglia [54]. PU.1 has been shown to have both transactivation and repression effects on different genes and cell types [40,55,56,57], potentially by interacting with different epigenetic histone modifiers [58] and transcription factors [59,60]. Currently, we are examining the mechanisms by which PU.1 and DB2313 regulate Cxcl9/10/11 expression in BMDMs, noting that these mechanisms may differ in microglia.

A crucial role for the CXCL9/10/11-CXCR3 chemokine axis in anti-tumor immunity has been demonstrated in various tumors [61,62], and high levels of CXCL9/10 are associated with increased infiltration of immune cells and better prognosis in melanoma patients [63]. Particularly, CXCL9/10/11 produced by TAMs are required for antitumor immune responses following immune checkpoint inhibitor treatments, as they play an essential role in recruiting CTLs and NK cells into tumors [53,64]. In line with these studies, we demonstrate that enhanced expression of CXCL9 by DB treatment was associated with increased infiltration of Th1 (CD4^+^/T-bet^+^), CTLs (CD8^+^/GrzB^+^), and NK (NK1.1^+^/GrzB^+^) into tumors (Figure 2), which was abrogated by CXCL9- and CXCR3-neutralizing antibodies (Figure 6B–D). Importantly, blocking the CXCR9-CXCR3 axis abolished both the overall tumor growth-suppressing effects (Figure 6A) and the overall immune-responsive transcriptomic changes in the tumors treated with CXCR3-neutralizing antibodies (Figure 7D) induced by DB, suggesting that the CXCR9-CXCR3 chemokine axis is a main driver of anti-tumor immune responses triggered by DB. Although the RNA-seq experiments did not detect changes, other CXCR3 ligands, including CXCL10 and 11, are not addressed in this study.

To date, enhancing the CXCL9/10/11-CXCR3 chemokine axis by administering recombinant CXCL9/10/11 proteins or using expression vector systems has demonstrated positive outcomes in various pre-clinical tumor models, including skin, lung, kidney, and colon tumors [65,66,67]. However, while the paracrine effects of chemokines released by macrophages recruit anti-tumor immune cells, the autocrine effects of the CXCL9/10/11-CXCR3 axis within tumor cells are linked to increased tumor growth and metastatic potential [68,69]. Therefore, selectively activating the CXCL9/10/11-CXCR3 paracrine axis is suggested to be a more effective anti-tumor strategy [53]. This study, for the first time, indicates that the pharmacological inhibition of PU.1 enhances the CXCL9-CXCR3 paracrine axis, likely by targeting TAMs. Given that the effectiveness of immune checkpoint inhibitors (such as PD-1/PD-L1 inhibitors) is partially limited by TAMs, and that the CXCL9/10/11-CXCR3 axis is crucial for their anti-tumor effects [70], PU.1 inhibition could serve as an effective combinatory strategy with immune checkpoint inhibitor therapies. This potential should be explored in further detailed studies.

In addition to macrophages, PU.1 is involved in early T cell development, where its expression is turned off at the thymic stage [71]. This dynamics in PU.1 expression is crucial for avoiding T cell malignancy [72]. Among mature T cells, PU.1 is uniquely expressed in helper T cell 9 (Th9) and Th2 cells [73]. IL-9-producing Th9 cells are a new subgroup of CD4^+^ T cells that are differentiated in response to TGF-β and IL-4 [74]. These cells have dual roles in tumorigenesis: on the one hand, they enhance the function of immunosuppressive regulatory T cells and promote tumor growth [75]; on the other hand, they suppress tumor growth by indirectly recruiting cytotoxic lymphocytes [76]. The effects of DB on Th9 cell function and its role in the anti-tumor response remain to be investigated. In contrast, Th2 cells also express PU.1, albeit at low levels, and knocking down PU.1 by siRNAs increases the production of Th2 cytokines [77,78,79]. We observed that DB-treated tumors showed no differences in the Th2 cell population from controls (Figure 2C), ruling out the involvement of Th2 cells. Of interest, DB substantially increased the population of Th1/Th2 hybrid cells while decreasing Treg cells (Figure 2C). Th1/Th2 hybrid T cells are known to naturally develop with intermediate phenotypes characteristic of both Th1 and Th2 cells [39,80]. While the role of Th1/Th2 hybrid T cells in tumor growth is unclear, the involvement of Treg cells in tumor progression is well established [81]. Intriguingly, Treg cells also express CXCR3 and can be recruited into tumors by CXCL9-producing BATF3^+^ dendritic cells [82]. We observed a substantial decrease in Treg cells in DB-treated tumors, likely due to the overriding inhibitory effects of DB on other cytokines/chemokines, such as IL-10 and CCLs, which are also required for generation and recruitment of Treg cells [81].

In addition, PU.1 plays a key role in early B cell development, likely up to the common lymphoid progenitor stage [83]. Although PU.1 continues to be expressed at low levels, it is dispensable for B cell differentiation [84,85]. However, PU.1 promotes the generation of B1 B cells [84] and plasma cells [86]. Plasma cells can infiltrate tumors, where they may confer anti-tumor effects by producing antibodies and presenting tumor antigens to T cells. However, they can also support tumor growth, likely by releasing immune-suppressive cytokines and ligands after being programmed to become immunosuppressive B cells [87]. We observed no differences in the populations of plasma and memory B cells (Figure 2F), ruling out their involvement in the anti-tumor effects of DB.

## 5. Conclusions

This study demonstrates that pharmacological inhibition of PU.1 suppresses tumor growth in melanoma and likely in breast cancer mouse models. This effect appears to be mediated, at least in part, by the selective recruitment of cytotoxic T lymphocytes (CTLs) and natural killer (NK) cells via the CXCL9–CXCR3 chemokine axis. These findings suggest a novel immunotherapeutic strategy that reprograms tumor-associated macrophages (TAMs) by targeting the transcription factor PU.1.

## Data Availability

All RNA-seq data are available in the Sequence Read Archive Accession numbers: PRJNA1191843 and PRJNA1191505.

## Supplementary Materials

The following supporting information can be downloaded at: https://www.mdpi.com/article/10.3390/cancers17162684/s1, Figure S1: Gating strategy for specific immune cell subtypes and representative flow cytometric plots; Figure S2: Depletion of macrophages by clodronate liposome; Figure S3: F4/80+ cell isolation efficiency from tumor tissue; Figure S4: PU.1/SPI1 is over-expressed in multiple cancers; Figure S5: Original images for Figure 5B; Table S1: List of antibodies used; Table S2: Differential gene expression list induced by DB in TAMs; Table S3: Enhanced functional gene nodes in tumor tissues.

## Abbreviations

The following abbreviations are used in this manuscript:

BMDM	Bone marrow-derived macrophages
DB	DB2313
CTL	Cytotoxic T lymphocytes
ETS	the E26 transformation-specific
LPS	Lipopolysaccharide
NK	Natural killer
TAM	Tumor-associated macrophage
TF	Transcription factor
Th1	T helper type 1
TME	Tumor microenvironment
Treg	Regulatory T
