# Supplementary material for: Inhibition of the Transcription Factor PU.1 Suppresses Tumor Growth in Mice by Promoting the Recruitment of Cytotoxic Lymphocytes Through the CXCL9-CXCR3 Axis
Source: Cancers (Basel). 2025 Aug 18;17(16):2684. doi: 10.3390/cancers17162684 (PMC12384184; doi:10.3390/cancers17162684)
Supplement: Supplementary file 1 [file cancers-17-02684-s001.zip › cancers-3801872-supplementary figures.pdf]

Supplementary Materials: Inhibition of the Transcription Factor PU.1 Suppresses Tumor Growth in Mice by Promoting the Recruitment of Cytotoxic Lymphocytes Through the CXCL9-CXCR3 Axis

Nichita Sleapnicov, Soon-Duck Ha, Shanshan Jenny Zhong, Jackie Duchscher, Sally Ezra, Shawn Shun-Cheng Li and Sung Ouk Kim

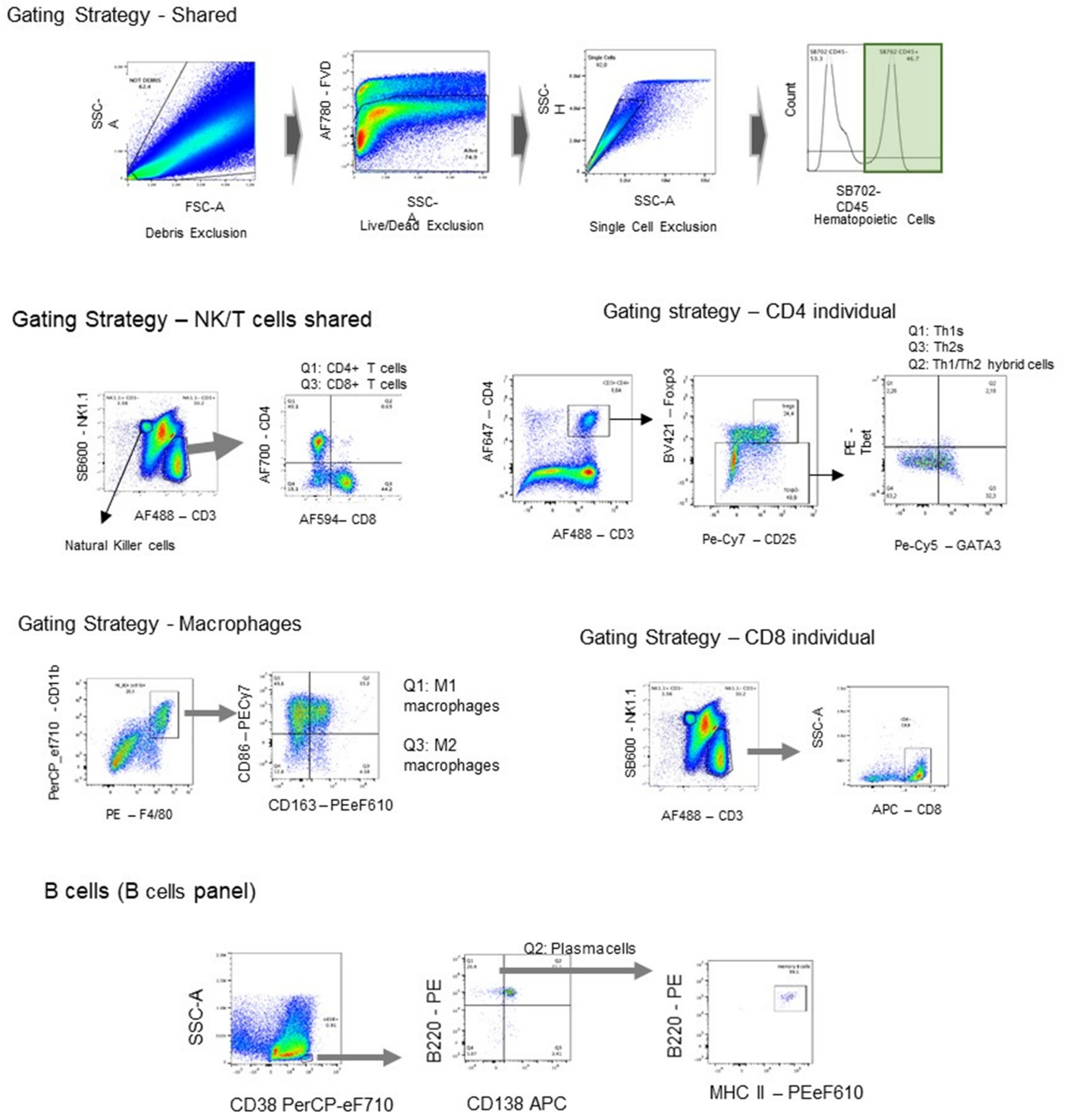

Figure S1. Gating strategy for specific immune cell subtypes and representative flow cytometric plots. Flow cytometry gating strategy for all cell types. All samples went through shared gates before cell-lineage sorting. T cells were either sorted with CD4/CD8 in one panel or with CD4 and CD8 in separate panels with subsets. NK cells were always phenotyped in the same panel with CD8+ T cells. B cells and macrophages were phenotyped in their own panels.

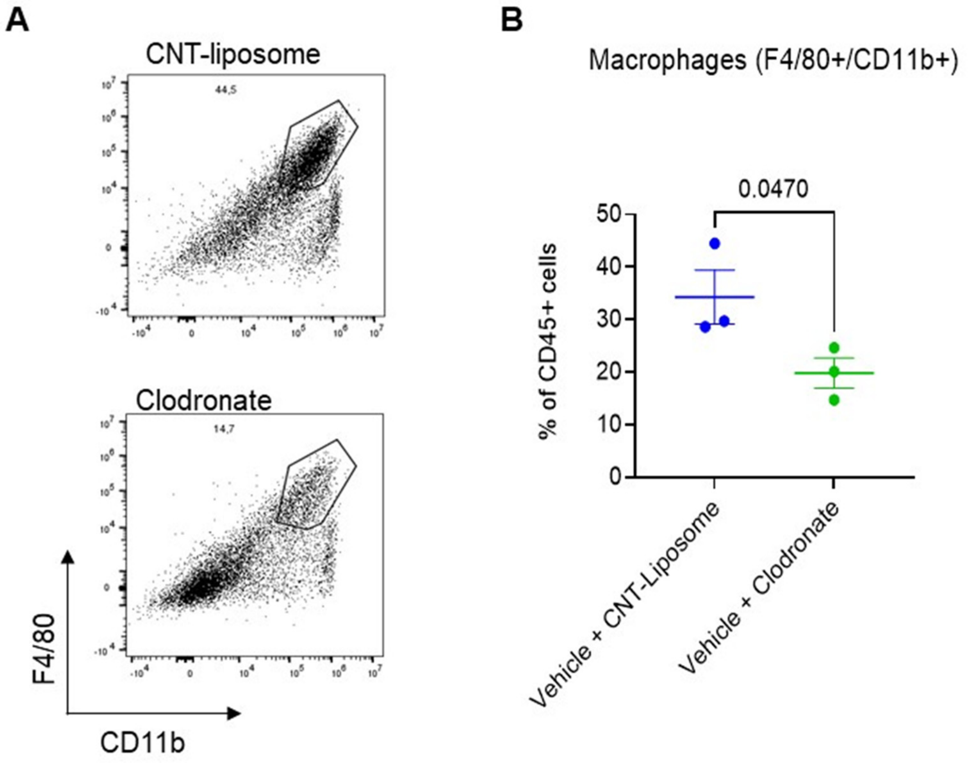

Figure S2. Depletion of macrophages by clodronate liposome. (**A**) FACS plots showing depletion of F4/80+/CD11b+ macrophages by clodronate gated on live, single CD45+ cells. B16-OVA bearing mice were injected with clodronate (1mg/mice) or control (CNT) liposome every 4 days for 2 weeks once tumors reached ~50~60 mm3.Tumors were homogenized and macrophage markers (F4/80, CD11b) were analyzed by flow cytometry (**B**) Relative frequencies of macrophages (CD45+/CD11b+/F4/80+) in control-liposome and clodronate-treated tumors. N = 3, statistical significance by unpaired student *t*-test.

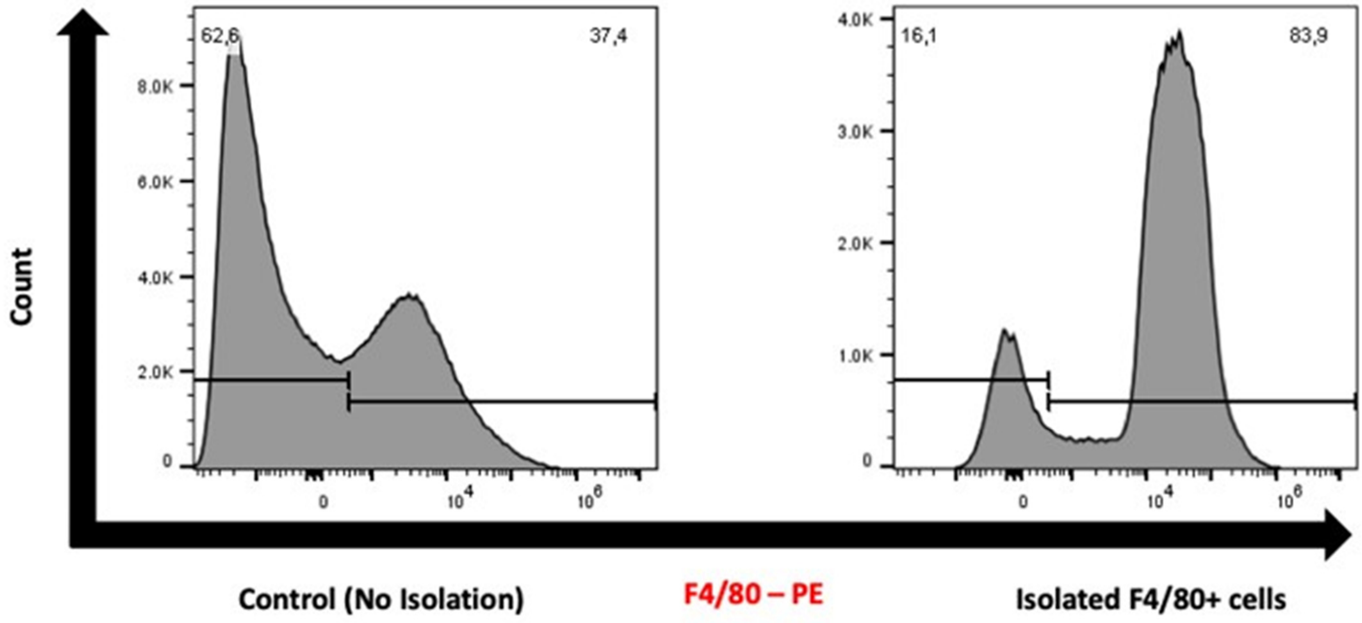

Figure S3. F4/80+ cell isolation efficiency from tumor tissue. F4/80+ macrophages were isolated from murine vehicle tumor cell suspensions by magnetic-activated cell sorting of cells positive for F4/80-PE antibody. Representative FACS plots shown of both tumor samples with no isolation and isolated F4/80+ cells, gated on single cells. PE-positive cells increased from 37.4% in control to 83.9% in isolated samples, with a more intense peak seen in the isolated sample.

A

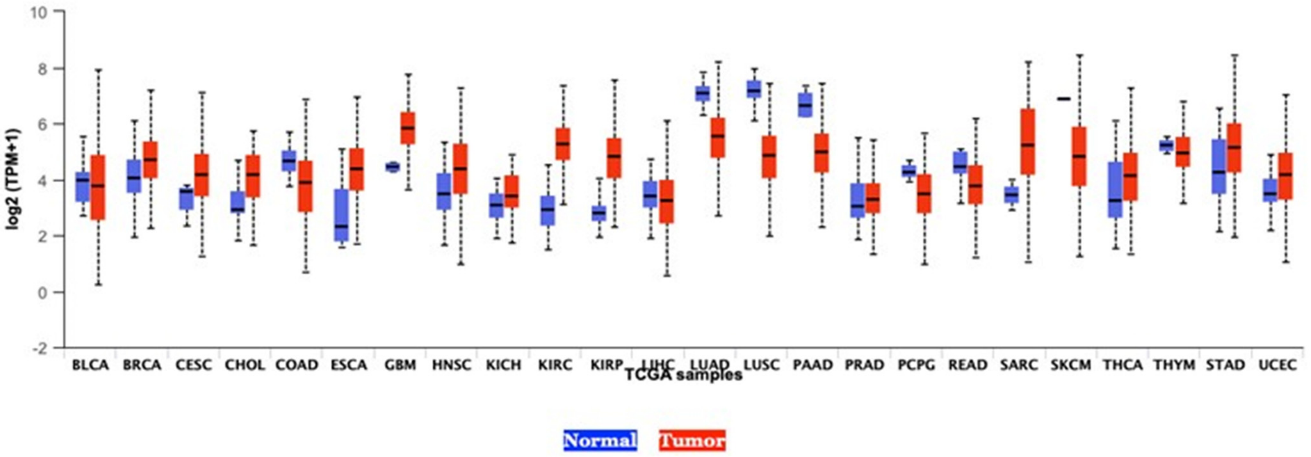

B

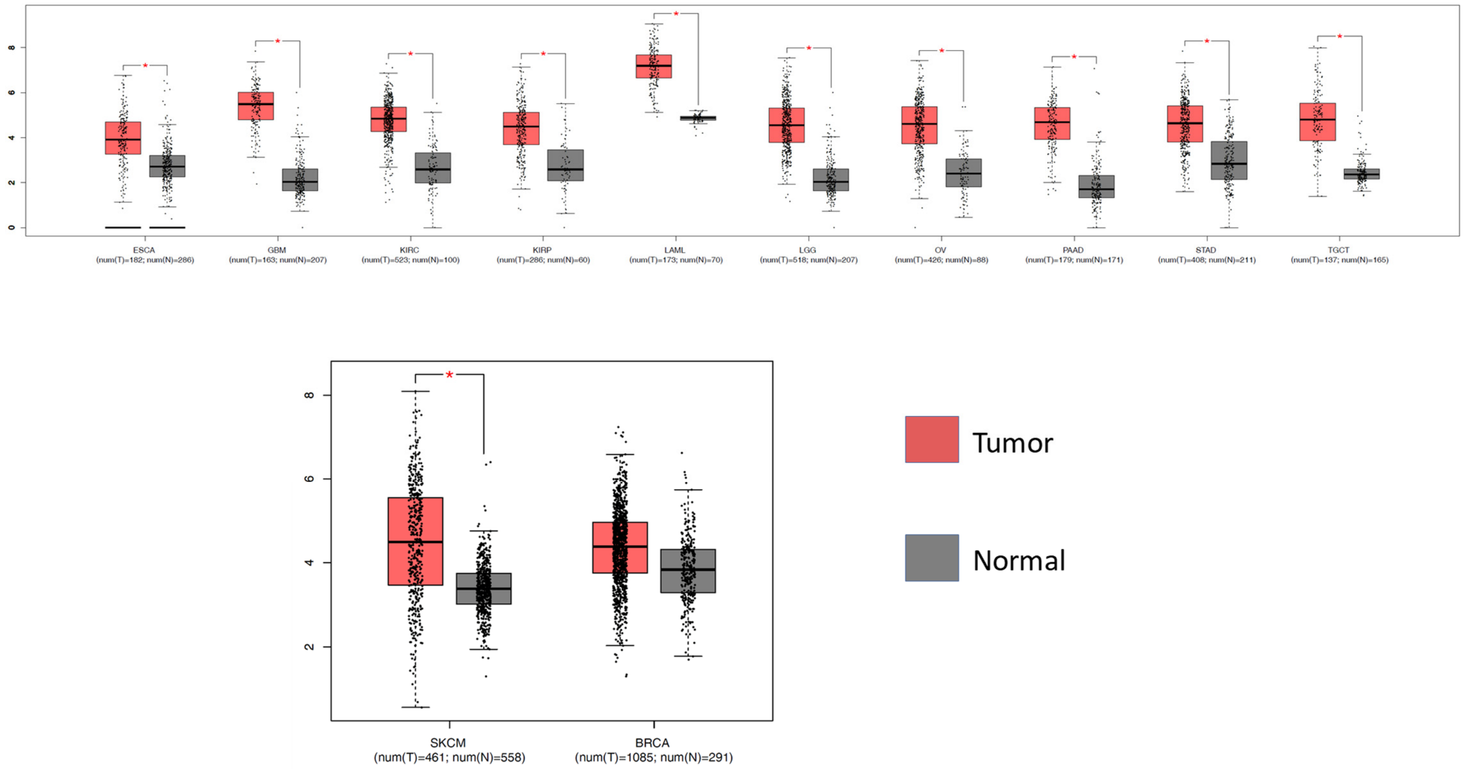

Figure S4. PU.1/SPI1 is over-expressed in multiple cancers. (**A**) Box plots showing the relative mRNA levels of SPI1 in tumors vs. adjacent normal tissues based on data from the TCGA. (**B**) SPI1 is significantly over-expressed in multiple cancers including melanoma (SKCM) and breast cancer (BRCA). *, $p < 0.01$, unpaired student t-test.

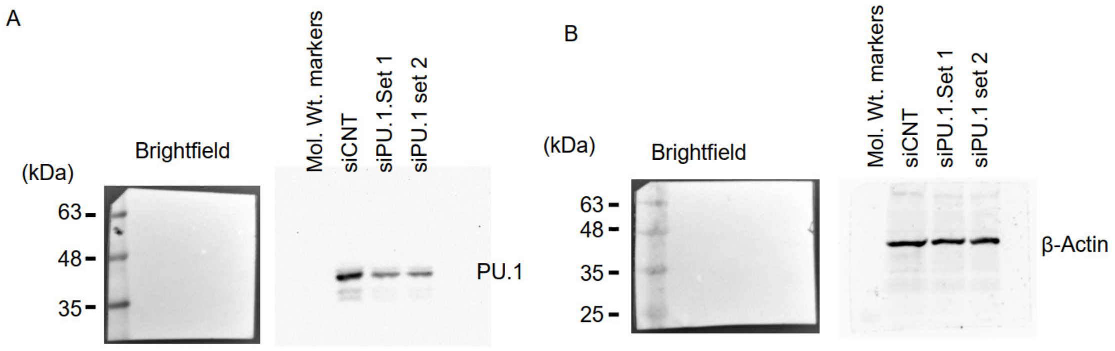

Figure S5. Original images for Fig. 5B. Western blot was were performed using PU.1 (**A**) and β-actin (**B**) antibodies. Western blot Images were developed using the chemiluminescence reagent (BioRad Clarity Max Western ECL system) and BioRad Chemidoc XR+ System. A & B. Left panels: An image captured using brightfield; right panels: A chemiluminescence images captured from PU.1 antibody-blotted membrane.
